# Early-onset preeclampsia predisposes to preclinical diastolic left ventricular dysfunction in the fifth decade of life: An observational study

**DOI:** 10.1371/journal.pone.0198908

**Published:** 2018-06-12

**Authors:** Anouk Bokslag, Constantijn Franssen, Lisa J. Alma, Igor Kovacevic, Floortje van Kesteren, Pim W. Teunissen, Otto Kamp, Wessel Ganzevoort, Peter L. Hordijk, Christianne J. M. de Groot, Walter J. Paulus

**Affiliations:** 1 Department of Obstetrics and Gynaecology, Amsterdam Cardiovascular Sciences, VU University Medical Center, Amsterdam, The Netherlands; 2 Department of Physiology, Amsterdam Cardiovascular Sciences, VU University Medical Center, Amsterdam, The Netherlands; 3 Department of Cardiology, Antwerp University Hospital, Antwerp, Belgium; 4 Department of Educational Development and Research, Faculty of Health, Medicine and Life Sciences, Maastricht University, Maastricht, The Netherlands; 5 Department of Cardiology, Amsterdam Cardiovascular Sciences, VU University Medical Center, Amsterdam, The Netherlands; 6 Department of Obstetrics and Gynaecology, Academic Medical Center, Amsterdam, The Netherlands; Universita degli Studi di Roma La Sapienza, ITALY

## Abstract

**Background:**

Systemic inflammation, endothelial dysfunction and deficient vascularization of either uterus or myocardium are mechanistic hallmarks of early-onset preeclampsia and heart failure with preserved ejection fraction (HFpEF). HFpEF is especially prevalent in elderly women and preceded in middle age by preclinical left ventricular (LV) diastolic dysfunction.

To detect if preeclampsia predisposes to HFpEF at later age, echocardiographic indices of LV function and of LV structure and biomarkers of systemic inflammation and of endothelial dysfunction were compared in middle-aged women with a history of early-onset preeclampsia or uncomplicated pregnancy.

**Methods and findings:**

Middle-aged women with a history of early-onset preeclampsia (n = 131) or uncomplicated pregnancy (n = 56) were prospectively recruited 9 to 16 years after pregnancy.

Women with a history of preeclampsia had higher body mass index (p = 0.006), blood pressure (p<0.001) and plasma levels of interleukin-6 (p = 0.005) and soluble intercellular adhesion molecule-1 (sICAM-1) (p = 0.014). They had thicker septal (p = 0.001) and posterior (p = 0.003) LV walls and worse diastolic LV function evident from reduced mean mitral annular lengthening velocity (E’mean; p = 0.007) and higher ratio of early diastolic mitral flow velocity (E) over E’mean (E/E’mean; p<0.001). Differences of sICAM-1, E’mean and E/E’mean remained significant after accounting for BMI and blood pressure.

**Conclusions:**

History of preeclampsia predisposes in middle age to worse LV diastolic function, which could increase the likelihood of later HFpEF development. This predisposition derives not only from persistent cardiovascular risk but may also be caused by persistent endothelial dysfunction hindering adequate vascularization in the uterus during pregnancy and in the myocardium in middle age.

## Introduction

Early-onset preeclampsia is currently attributed to generalized maternal endothelial dysfunction mainly evident from arterial hypertension and proteinuria, which are respectively induced by disturbed vascular reactivity and glomerular leakage[1]. Maternal endothelial dysfunction is presumed to result from placental release of antiangiogenic factors like sFlt-1, which counteract proangiogenic factors like PIGF in transforming small caliber uterine spiral vessels into large caliber capacitance vessels necessary to maintain normal placental function[2]. In accordance with this deranged equilibrium between antiangiogenic and proangiogenic factors, relative concentrations of sFlt-1 and PIGF were recently suggested to predict evolving preeclampsia[3]. Upstream to the placental release of antiangiogenic factors are among others maternal systemic inflammation evident from placental infiltration with macrophages and maternal oxidative stress evident from raised plasma levels of oxidative stress biomarkers[4, 5].

Preeclampsia also portends prevalence of cardiovascular risk and diseases later in life[6–8]. The latter was obvious from a recent meta-analysis involving 258 000 women with preeclampsia and identifying a 4-fold increase in incident heart failure and a 2-fold increase in incident coronary artery disease[7]. Higher incidence of heart failure than coronary disease, suggests an important contribution of heart failure with preserved ejection fraction (HFpEF) to the incident heart failure. HFpEF is especially prevalent in women without coronary artery disease, features normal overall systolic left ventricular (LV) function but abnormal diastolic LV function and is preceded in middle age by preclinical LV diastolic dysfunction[9, 10]. An emerging paradigm for HFpEF suggests HFpEF to be driven by a signalling cascade sharing many features with preeclampsia as it involves metabolic risk, systemic inflammation, endothelial dysfunction, oxidative stress and myocardial infiltration by macrophages[11–13]. The mechanistic similarity between HFpEF and preeclampsia is supported by plasma biomarker profiles which resemble one another in both conditions[14] and the persistence after pregnancy of endothelial dysfunction in women with a history of preeclampsia[15].

HFpEF is preceded in middle age by preclinical LV diastolic dysfunction[9, 10, 16]. Several echocardiographic studies also reported on preclinical LV dysfunction in patients with early-onset preeclampsia. During pregnancy, patients with early-onset preeclampsia had higher LV mass, larger LV volumes and lower LV ejection fraction (LVEF) than normotensive pregnant and non-pregnant women[17]. These findings were subsequently implemented with evidence of diastolic LV dysfunction[18–20] and worse myocardial strain on speckle tracking echocardiograms[21]. Findings during pregnancy were more severe in early onset preeclampsia than in term preeclampsia[22] and paralleled by increases in plasma natriuretic peptide levels[17, 18, 23]. Postpartum persistence of these abnormalities as preclinical LV dysfunction or LV hypertrophy remains unclear. In a small cohort of 14 women reassessed after 13 to 18 years, systolic LV function remained depressed but diastolic LV function was normalized[24]. A similar depression of systolic LV function was observed two years postpartum in two cohorts consisting respectively of 64 and 30 patients[25, 26] and four to ten years postpartum in a cohort comprising 107 patients[27]. The latter study also observed a high prevalence (67%) of persistent concentric LV remodelling. A recent study however failed to observe remaining alterations of systolic LV function, diastolic LV function or LV wall thickness in a small cohort of 15 patients 11 years after pregnancy[28]. The discrepant outcome of these studies relates to the limited time span of follow-up, the small size of the cohorts and correction for confounding cardiovascular risk factors.

Because of epidemiological and mechanistic links between preeclampsia and HFpEF and the uncertainty of the long-term persistence of the acute effects of preeclampsia on LV remodelling, the present study compared clinical characteristics, echocardiographic systolic and diastolic LV function and biomarker profiles in women with a history of early-onset preeclampsia and uncomplicated pregnancy. In contrast to previous studies, a large cohort of early-onset preeclampsia patients (n = 131) was investigated 9 to 16 years after their index pregnancy, in the fifth decade of life when preclinical diastolic LV dysfunction is known to become manifest[16]. Furthermore, at the time of reassessment echocardiographic findings and biomarker profiles were adjusted for confounding cardiovascular risk factors.

## Methods

### Study population

The study population was previously described[8]. From two tertiary medical centres in the Netherlands, all medical records from 1998 to 2005 of women with early-onset preeclampsia were screened consecutively and all eligible women were invited. Participating women with a history of early-onset preeclampsia were matched with women with a history of an uncomplicated pregnancy for maternal age (range ± 5 years) and date of delivery (range ± 1 year). Women with early-onset preeclampsia delivered before 34 weeks gestation, had blood pressure ≥140/90 mmHg and proteinuria ≥300 mg/24h in accordance to the criteria used at the time of preeclampsia diagnosis[29]. Women with an uncomplicated pregnancy delivered at term (≥37 weeks gestation), after a normotensive pregnancy, without intrauterine growth restriction of the neonate. For both women with a history of early-onset preeclampsia and uncomplicated pregnancy, exclusion criteria were: hypertension before index pregnancy or in first trimester; use of antihypertensive medication before index pregnancy; diabetes mellitus or gestational diabetes in index pregnancy; cardiovascular diseases and use of cardiovascular medication before index pregnancy; multiple pregnancy; fetal congenital abnormalities in index pregnancy; pregnant during risk assessment or within six months before risk assessment; breastfeeding during risk assessment.

All participants gave written informed consent and were screened in the VU University Medical Center. Data were collected between 2014 and 2016. Approval for the study was obtained from the medical ethics committee of the VU University Medical Center in Amsterdam and from the hospital board of the Academic Medical Center Amsterdam (protocol approval: NL38972.029.12; Dutch trial registration: NTR5297).

### Patient characteristics

Assessment of patient characteristics was previously described[8] and consisted of a questionnaire addressing personal and family medical history, educational level, blood pressure measurement and anthropometrics. Hypertension was defined as either current use of antihypertensive medication and/or blood pressure ≥140/90 mmHg measured at risk assessment[30]. Metabolic syndrome was diagnosed according to the Adult Treatment Panel III criteria[31]. Educational level was subdivided in low (primary school, lower vocational training, pre-vocational secondary education), intermediate (secondary vocational education, senior general secondary education, pre university education,) and high (higher professional education, university education).

### Echocardiography

Cardiac function was assessed by transthoracic ultrasound using a Philips X5-1 transducer on a Philips IE-33 cardiac ultrasound system. Both sonographer and attending cardiologist were blinded for pregnancy history at the time of the echocardiographic examination. Septal wall thickness, posterior wall thickness and LV mass were measured with M-mode on parasternal long axis views and LV mass was indexed by body surface area (LV mass index). LV volumes and ejection fraction were measured by biplane method of disks summation. Early (E) and late (A) diastolic mitral flow velocity and deceleration time of the mitral valve (MV dec time) were measured, and E/A ratio was calculated. Left atrial volume was measured by disks summation algorithm and indexed by body surface area (LA volume index). Mitral annular lengthening velocities (E′) were measured by tissue Doppler imaging on the lateral and septal part of the mitral valve annulus. From these values, the average E′ (E’mean) and E/E’mean ratio were calculated.

### Biomarker analysis

Two endothelial activation markers, soluble intercellular adhesion molecule-1 (sICAM-1) and soluble endothelial selectin (sE-selectin), were measured with ELISA-sets (R&D Systems, catalogue numbers DY724 and DY720 respectively). Experiments were carried out according to the standard protocol of the kit. Plasma samples were diluted 1:10 for sE-selectin and 1:1000 for sICAM-1. The levels of three inflammatory cytokines, interleukin 6 (IL-6), tumor necrosis factor alpha (TNF-α), and high-sensitivity c-reactive protein (hsCRP) were measured according to standardized protocols.

### Statistical analysis

Normally distributed numerical data were reported as means with standard deviations, not normally distributed numerical data as medians with interquartile ranges and categorical data as percentages. Differences were analysed by unpaired t-test, Mann-Whitney U test and Fisher’s exact test when appropriate. Multivariate linear regression analyses were used to study the relationship between a history of early-onset preeclampsia and outcome variables, in combination with potential confounding factors including age, smoking status, blood pressure, body mass index (BMI) and educational level. When comparing multiple groups, two-way ANOVA and Kruskal-Wallis tests were used for normally and not normally distributed variables respectively. Bonferroni post hoc tests were performed to analyse differences between individual groups. To calculate the correlation between biomarkers and nonparametric variables, Spearman’s rank correlation was used. In all analyses, a p-value <0.05 was considered statistically significant. Data were analysed using SPSS 22 software (Chicago, IL).

## Results

### Clinical characteristics

**Fig 1:**
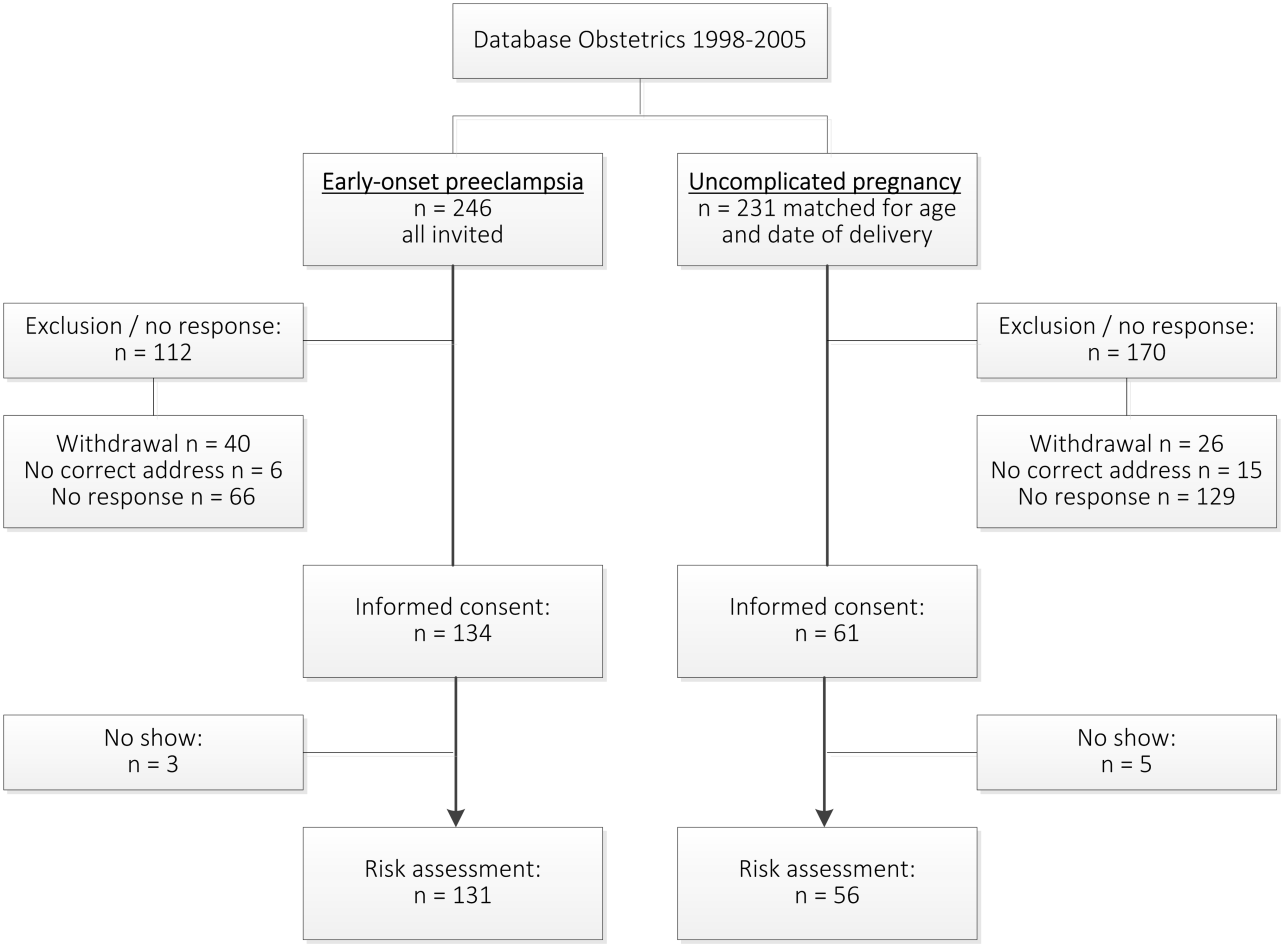
Recruitment study population.

Women with a history of early-onset preeclampsia (n = 131) were compared to women with a history of uncomplicated pregnancy (n = 56) (Fig 1). Women with a history of early-onset preeclampsia were slightly younger and interval between index pregnancy and risk assessment was shorter (Table 1). Women with a history of early-onset preeclampsia had higher systolic, diastolic and mean arterial blood pressures and a higher prevalence of arterial hypertension. Women with former preeclampsia also had higher BMI, larger waist circumference and a higher prevalence of metabolic syndrome. Among them, two women had increased glucose levels (fasting glucose >7 mmol/ml) and one had impairment in glomerular filtration rate (eGFR <60 mL/min/1.73m2).

**Table 1:** Baseline characteristics in the fifth decade of life.

Characteristics		Uncomplicated pregnancy n = 56	Early-onset preeclampsia n = 131	p-value
Age, years	mean ± SD	46.5 ± 4.8	44.0 ± 5.6	**0.004**
Post index pregnancy, years	mean ± SD	14.2 ±2.3	13.1 ±2.2	**0.003**
Caucasian	n (%)	49 (87.5)	113 (86.3)	1.000
Current smoking	n (%)	9 (16.1)	23 (17.6)	1.000
Educational level				0.081
Low	n (%)	6 (10.7)	31 (23.7)	
Intermediate	n (%)	19 (33.9)	45 (34.4)	
High	n (%)	31 (55.4)	54 (41.2)	
Family history*				
MI <60 years	n (%)	11 (19.6)	24 (18.3)	0.895
Stroke <60 years	n (%)	2 (3.6)	10 (7.6)	0.095
**Blood pressure**				
SBP, mmHg	mean ± SD	115 ± 17.0	126 ± 18.6	**<0.001**
DBP, mmHg	mean ± SD	74 ± 9.4	82 ± 9.8	**<0.001**
MAP, mmHg	mean ± SD	87.4 ± 11.3	96.7 ± 11.8	**<0.001**
**Anthropometrics**				
BMI, kg/m^2^	median [IQR]	23.9 [21.2─26.6]	25.6 [22.7─28.9]	**0.006**
Waist circumference, cm	median [IQR]	77.0 [71.0─82.8]	79.0 [75.0─90.0]	**0.007**
**Risk profile**				
Hypertension†	n (%)	8 (14.3)	50 (38.2)	**0.001**
Metabolic syndrome	n (%)	1 (1.8)	22 (16.8)	**0.003**

MI = myocardial infarction; SBP = systolic blood pressure; DBP = diastolic blood pressure; MAP = mean arterial pressure; BMI = body mass index.

* Only first degree relatives reported.

^†^ Current use of antihypertensive medication and/or blood pressure ≥140/90 mmHg at risk assessment.

### Echocardiography

**Fig 2:**
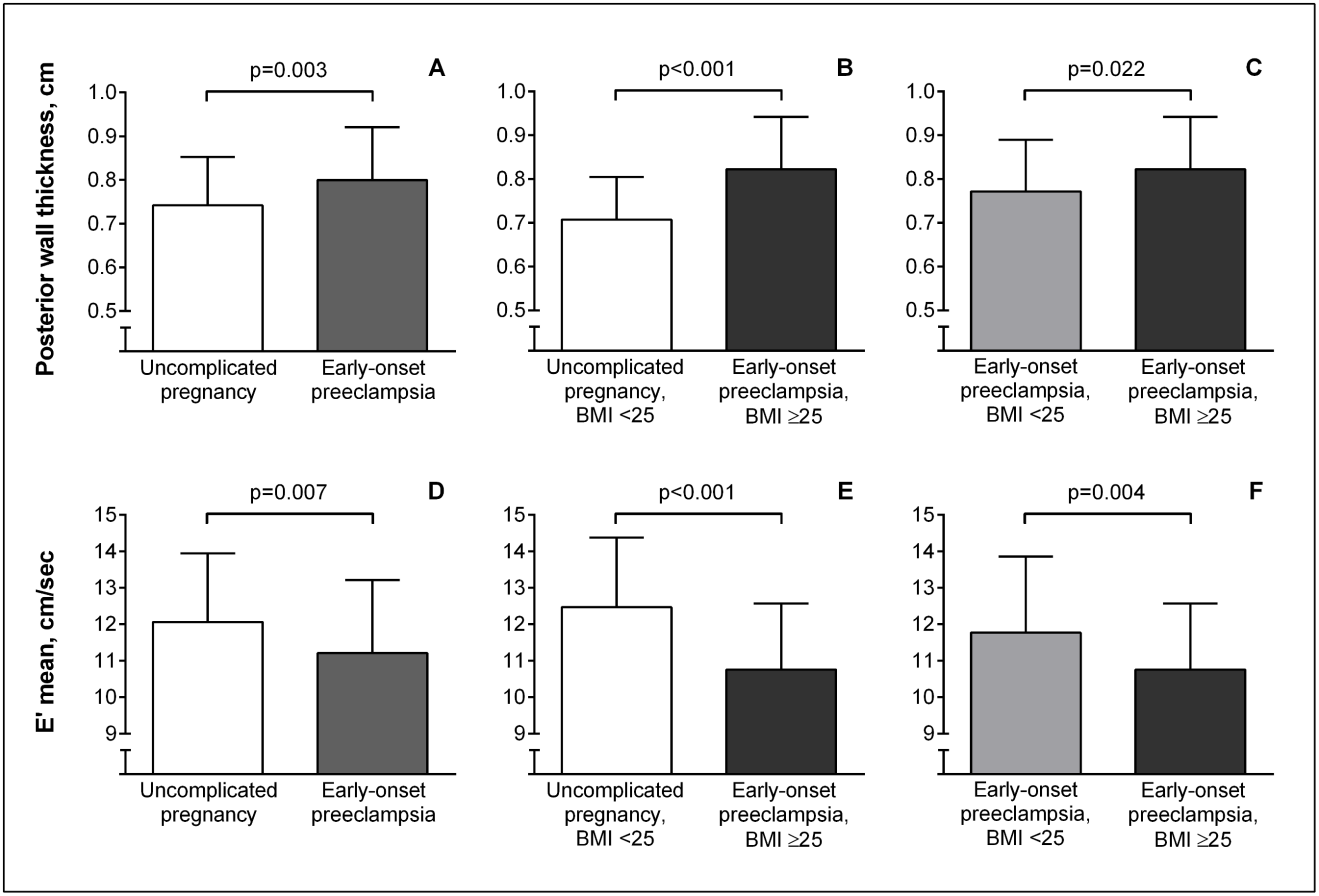
Effect of pregnancy history on posterior wall thickness and E’mean. A. Effect of pregnancy history on posterior wall thickness; B. Enhanced effect of pregnancy history on posterior wall thickness when comparing patients with uncomplicated pregnancy and BMI<25 kg/m^2^ to patients with preeclampsia and BMI ≥25 kg/m^2^; C. Effect of persistent metabolic risk (BMI ≥25 kg/m^2^) on posterior wall thickness in women with a history of preeclampsia; D. Effect of pregnancy history on E’mean; E. Enhanced effect of pregnancy history on E’mean when comparing patients with uncomplicated pregnancy and BMI<25 kg/m^2^ to patients with preeclampsia and BMI ≥25 kg/m^2^; F. Effect of persistent metabolic risk (BMI ≥25 kg/m^2^) on E’mean in women with a history of preeclampsia.

In their fifth decade of life, several echocardiographic measures of LV structure and LV diastolic function differed between women with a history of early-onset preeclampsia and women with a history of uncomplicated pregnancy (Table 2). Septal wall thickness, posterior wall thickness (Fig 2A) and LV mass index were higher in women with a history of early-onset preeclampsia while LV end diastolic volume index and left atrial volume index remained comparable in both groups.

**Table 2:** Echocardiographic indices and biomarkers in patients with uncomplicated pregnancy versus early-onset preeclampsia.

Characteristics		Uncomplicated pregnancy n = 56	Early-onset preeclampsia n = 131	p-value
**Echocardiography**				
Septal wall thickness, cm	mean ± SD	0.73 ± 0.11	0.79 ± 0.12	**0.001**
Posterior wall thickness, cm	mean ± SD	0.74 ± 0.11	0.80 ± 0.12	**0.003**
LV mass index, gr/m^2^	mean ± SD	60.5 ± 13.1	65.4 ± 14.7	**0.035**
LV end diastolic volume index, ml/m^2^	mean ± SD	57.0 ± 13.1	56.4 ± 15.4	0.800
Ejection fraction, %	mean ± SD	58.4 ± 5.5	59.1 ± 6.3	0.498
E, cm/sec	mean ± SD	81.63 ± 12.51	85.86 ± 15.64	0.075
A, cm/sec	mean ± SD	59.85 ± 12.97	65.36 ± 16.25	**0.026**
E/A ratio	median [IQR]	1.27 [1.12–1.67]	1.25 [1.08–1.54]	0.250
MV dec time, sec	median [IQR]	0.20 [0.18–0.21]	0.19 [0.18–0.21]	0.490
LA volume index, ml/m^2^	mean ± SD	23.4 ± 6.71	22.9 ± 6.40	0.633
E’lateral, cm/sec	mean ± SD	13.26 ± 2.55	12.52 ± 2.57	0.073
E’septal, cm/sec	mean ± SD	10.87 ± 1.87	9.94 ± 2.00	**0.004**
E’mean, cm/sec	mean ± SD	12.06 ± 1.88	11.21 ± 2.00	**0.007**
E/E’ mean ratio	mean ± SD	6.86 ± 1.16	7.86 ± 1.95	**<0.001**
**Biomarkers**				
IL-6, pg/ml	mean ± SD	0.46 ± 0.23	0.64 ± 0.61	**0.005**
sICAM-1, ng/ml	mean ± SD	495 ± 246	601 ± 274	**0.014**
sE-selectin, ng/ml	mean ± SD	8.22 ± 2.88	8.16 ± 3.36	0.915
TNF-α, pg/ml	mean ± SD	2.03 ± 0.69	1.90 ± 0.56	0.179
hsCRP, mg/l	mean ± SD	2.07 ± 3.34	3.35 ± 5.43	0.108

LV = left ventricular; E = early diastolic mitral flow velocity; A = late diastolic mitral flow velocity; MV dec time = mitral valve deceleration time; LA = left atrial; E’ = mitral annular lengthening velocity; IL-6 = interleukin 6; sICAM-1 = soluble intercellular adhesion molecule-1; sE-selectin = soluble endothelial selectin; TNF-α = tumor necrosis factor alpha; hsCRP = high-sensitivity c-reactive protein

Several indices of LV filling dynamics differed between women with a history of early-onset preeclampsia and women with a history of uncomplicated pregnancy. Septal mitral annular lengthening velocity (E’septal) and the mean of the septal and lateral E’ (E’mean) (Fig 2D) were lower. The ratio of early diastolic mitral flow velocity (E) over E’mean (E/E’mean) and late diastolic mitral flow velocity (A) were higher. LV ejection fraction was comparable in both groups. In multivariate regression analysis accounting for confounders including age, smoking status, systolic blood pressure, BMI and educational level, E’mean remained significantly lower and E/E’mean significantly higher in women with a history of early-onset preeclampsia compared to women with a history of uncomplicated pregnancy (Table 3). When preclinical diastolic LV dysfunction was defined by an E/E’ ratio exceeding age and gender adjusted reference values by more than 1SD [32, 33], 19.1% of women with a history of preeclampsia had preclinical diastolic LV dysfunction compared to 5.4% of women with a history of uncomplicated pregnancy (p = 0.014).

**Table 3:** Multivariate linear regression analysis of a history of early-onset preeclampsia on diastolic function.

Dependent variable	B		95% confidence interval		p-value*
		Beta	Lower bound	Upper bound	
**Echocardiography**					
Septal wall thickness	0.030	0.117	-0.006	0.067	0.103
Posterior wall thickness	0.021	0.081	-0.016	0.057	0.261
LV mass index	0.469	0.015	0.200	0.842	0.842
E’mean	-0.665	-0.153	-1.242	-0.088	**0.024**
E/E’mean ratio	0.829	0.212	0.264	1.394	**0.004**
**Biomarkers**					
IL-6	0.152	0.132	-0.022	0.326	0.086
sICAM-1	88.881	0.151	3.059	174.703	**0.042**
sE-selectin	-0.758	-0.113	-1.841	0.271	0.144
TNF-α	-0.164	-0.125	-0.360	0.033	0.103
hsCRP	0.354	0.033	-1.234	1.942	0.661

E’ = mitral annular lengthening velocity; E = early diastolic mitral flow velocity; IL-6 = interleukin 6; sICAM-1 = soluble intercellular adhesion molecule-1; sE-selectin = soluble endothelial selectin; TNF-α = tumor necrosis factor alpha; hsCRP = high-sensitivity c-reactive protein;

* Dependent variables were adjusted for age, smoking status, systolic blood pressure, body mass index and educational level

To further explore the confounding effect of persistent metabolic risk, the population was split up into 4 groups in accordance to pregnancy history and BMI at follow-up 9 to 16 years after index pregnancy (Table 4). Differences observed between women with uncomplicated pregnancy and BMI <25 kg/m^2^ and women with history of preeclampsia and BMI ≥25 kg/m^2^ exceeded the differences observed in the total population (Table 2) for septal wall thickness (p<0.001), posterior wall thickness (p<0.001, Fig 2B), LV mass index (p = 0.01), E’lateral (p = 0.05), E’septal (p<0.001), E’mean (p<0.001, Fig 2E) and E/E’mean (p = 0.004). Effects of persistent metabolic risk became evident on post hoc analysis and consisted of increased posterior wall thickness (p = 0.02, Fig 2C), smaller E’septal (p = 0.02) and E’mean (p = 0.004, Fig 2F) in women with a history of preeclampsia and BMI ≥25 kg/m^2^ compared to women with a history of preeclampsia and BMI <25 kg/m^2^. Effects of preeclampsia at matched metabolic risk became also evident on post hoc analysis and consisted of thicker septal (p = 0.02) and posterior (p = 0.04) walls in women with a history of preeclampsia and BMI <25 kg/m^2^ compared to women with uncomplicated pregnancy and BMI <25 kg/m^2^ and of higher E/E’mean (p = 0.02) in women with a history of preeclampsia and BMI ≥25 kg/m^2^ compared to women with uncomplicated pregnancy and BMI ≥25 kg/m^2^.

**Table 4:** Results: Subgroups based on pregnancy history and BMI in the fifth decade of life.

Characteristics		Uncomplicated pregnancy, BMI <25 n = 32	Uncomplicated pregnancy, BMI ≥25 n = 24	Early-onset preeclampsia, BMI <25 n = 60	Early-onset preeclampsia, BMI ≥25 n = 71	p-value*
Septal wall thickness, cm	mean ± SD	0.70 ± 0.10	0.76 ± 0.11	0.76 ± 0.11	0.81 ± 0.12	**<0.001**
Posterior wall thickness, cm	mean ± SD	0.71 ± 0.10	0.79 ± 0.11	0.77 ± 0.12	0.82 ± 0.12	**<0.001**
LV mass index, gr/m^2^	mean ± SD	57.6 ± 11.0	64.3 ± 14.8	63.1 ± 12.6	67.4 ± 16.0	**0.014**
LV end diastolic volume index, ml/m^2^	mean ± SD	56.4 ± 12.7	57.9 ± 13.9	54.2 ± 12.0	58.3 ± 17.6	0.375
Ejection fraction, %	mean ± SD	58.3 ± 4.9	58.5 ± 6.4	59.0 ± 5.8	59.1 ± 6.8	0.822
E, cm/sec	mean ± SD	83.23 ± 12.84	79.49 ± 11.92	86.26 ± 14.59	85.53 ± 16.55	0.250
A, cm/sec	mean ± SD	58.46 ± 14.70	61.70 ± 10.22	63.91 ± 17.36	66.59 ± 15.27	0.088
E/A ratio	median [IQR]	1.51 [1.12–1.78]	1.22 [1.12–1.49]	1.30 [1.08–1.67]	1.24 [1.05–1.43]	0.121
MV dec time, sec	median [IQR]	0.19 [0.18–0.21]	0.20 [0.19–0.22]	0.19 [0.18–0.21]	0.19 [0.18–0.21]	0.561
LA volume index, ml/m^2^	mean ± SD	23.7 ± 6.41	22.9 ± 7.24	22.4 ± 6.68	23.2 ± 6.20	0.816
E’lateral, cm/sec	mean ± SD	13.62 ± 2.50	12.78 ± 2.59	13.09 ± 2.65	12.05 ± 2.41	**0.018**
E’septal, cm/sec	mean ± SD	11.33 ± 1.80	10.25 ± 1.82	10.52 ± 2.09	9.46 ± 1.80	**<0.001**
E’mean, cm/sec	mean ± SD	12.47 ± 1.91	11.52 ± 1.73	11.77 ± 2.09	10.75 ± 1.81	**<0.001**
E/E’mean ratio	mean ± SD	6.79 ± 1.29	6.96 ± 0.97	7.50 ± 1.59	8.16 ± 2.17	**<0.001**

BMI = body mass index, measured at risk assessment 9–16 years after pregnancy; LV = left ventricular; E = early diastolic mitral flow velocity; A = late diastolic mitral flow velocity; MV dec time = mitral valve deceleration time; LA = left atrial; E’ = mitral annular lengthening velocity.

* comparing the four subgroups by two-way ANOVA or Kruskal-Wallis tests when appropriate.

### Biomarkers

Plasma level of IL-6 was higher in women with a history of early-onset preeclampsia than in women with a history of uncomplicated pregnancy (Table 2). Higher level of IL-6 related to several features of metabolic syndrome including waist circumference (r = .257; p = 0.01), BMI (r = .272; p = 0.01), fasting triglycerides (r = .155; p = 0.05) and fasting HDL cholesterol (r = -.199; p = 0.01). Women with a history of early-onset preeclampsia also had a higher plasma level of sICAM-1. This higher level of sICAM-1 was related to features of metabolic syndrome such as waist circumference (r = .188; p = 0.05), BMI (r = .166; p = 0.05), fasting triglycerides (r = .211; p = 0.01) and fasting HDL cholesterol (r = -.175; p = 0.05). In multivariate regression analysis accounting for confounders including age, smoking status, systolic blood pressure, BMI, and educational level, sICAM-1 remained significantly higher in women with a history of early-onset preeclampsia compared to women with a history of uncomplicated pregnancy (Table 3). N Terminal pro Brain Natriuretic Peptide (NT-proBNP) levels were comparable in women with and without a history of preeclampsia (51[28–78] vs. 52[31–86] ng/L; mean [IQR]; p = 0.57).

## Discussion

The current study observed worse diastolic LV function and higher prevalence of preclinical diastolic LV dysfunction in women in the fifth decade of life with a history of early-onset preeclampsia as compared to women with a history of uncomplicated pregnancy. Worse diastolic LV function coincided with higher plasma levels of IL-6 and sICAM-1, which respectively reflected systemic inflammation and endothelial activation.

The present study differed from previous investigations assessing persistence of preeclampsia-induced LV remodelling by the size of the cohort (n = 131), the length of follow-up (9 to 16 years) and the adjustment of outcomes for cardiovascular risk factors such as age, smoking, blood pressure, BMI and educational level. In the present study, worse diastolic LV function was evident not only from comparison with women with an uncomplicated pregnancy but also from comparison with age and gender adjusted reference values[32].

Although diastolic LV dysfunction corresponded to the preclinical stage, it implies an increased risk of heart failure development. A recent meta-analysis found that a 1SD increase in E/E’ ratio exceeding age and gender adjusted reference values, significantly enhances the risk for incident heart failure (RR:1.20; 95% CI: 1.08–1.33)[33]. In women with a history of preeclampsia, 19.1% had a 1 SD increase of the E/E’ ratio, compared to 5.4% of women with a history of uncomplicated pregnancy. The present study did not observe patients who had evolved to clinically overt or stage C heart failure as evident from the comparable NT-proBNP values. Failure to observe patients with overt HFpEF probably resulted from the limited time span of postpartum follow-up. In the presence of preclinical diastolic LV dysfunction, the risk for evolution from preclinical diastolic LV dysfunction or stage B heart failure to stage C heart failure is however substantial and estimated at 11.6% over a three year time span[34]. In the patients with a history of preeclampsia, preclinical diastolic LV function was accompanied by concentric LV remodelling evident from increased LV mass index at unaltered LV end-diastolic volume index. A high prevalence of concentric LV remodelling (67%) was recently also reported by another study in a relatively large cohort (n = 107) of women with a history of preeclampsia four to ten years postpartum[27]. A trend for more concentric LV remodelling persists when preeclampsia and peripartum cardiomyopathy coexist[35]. In the presence of coexisting preeclampsia, patients with peripartum cardiomyopathy had smaller LV cavity, larger LV wall thickness and lower incidence of eccentric remodelling with recovery of LV ejection fraction occurring in 80% of patients versus 25% of patients without preeclampsia[36].

In the present study, a history of early-onset preeclampsia predisposed to worse diastolic LV function later in life even after correction for cardiovascular risk factors such as age, smoking, blood pressure, BMI and educational level (Table 3). These corrections are relevant not only to a direct effect of a history of preeclampsia but are also important for potential selection bias. Willingness to participate might be higher in women who had a reason to be examined or reassured on their cardiovascular status, for example because of positive family history of myocardial infarction, high BMI or smoking. This however applied to both groups of participants and did therefore not affect the observed differences.

HFpEF patient populations usually have an average BMI of ±30kg/m^2^ consistent with obesity. In the present study, the patients with a history of preeclampsia had higher BMI than controls and had already passed the threshold value for overweight (25 kg/m^2^). When patients were split up into four groups in accordance to pregnancy history and BMI larger or smaller than 25 kg/m^2^, effects of pregnancy history independent of BMI became evident and consisted of higher E/E’mean in women with a history of preeclampsia and BMI ≥25 kg/m^2^ compared to women with uncomplicated pregnancy and BMI ≥25 kg/m^2^. This finding supports the postpartum effects of preeclampsia to result not only from persistent cardiovascular risk, but possibly also from a primary inability to provide adequate tissue vascularization with insufficient utero-placental vascularization contributing to preeclampsia at the time of pregnancy and insufficient myocardial vascularization contributing to diastolic LV dysfunction in the fifth decade of life[10–13, 37]. The latter is consistent with a recently proposed paradigm whereby comorbidities, and especially metabolic comorbidities, trigger a systemic inflammatory state that results in coronary microvascular endothelial dysfunction, which alters paracrine signalling between endothelial cells and cardiomyocytes and allows leucocytes to infiltrate the myocardium. Altered paracrine signalling results in low myocardial nitric oxide and cyclic guanosine monophosphate content, which stiffens cardiomyocytes and removes the brake on cardiomyocyte hypertrophy, whereas leucocyte infiltration leads to activation of myofibroblasts and interstitial collagen deposition. This recently proposed paradigm for HFpEF is supported by a distinct biomarker profile and by evidence of inadequate tissue vascularization. HFpEF patients have elevated markers of systemic inflammation and endothelial activation,[38–41] which correlate with echocardiographic indices of diastolic LV dysfunction[42]. These elevated markers were also found in our patients with a history of preeclampsia, with higher levels of IL-6 and sICAM-1. This was likely also induced by metabolic comorbidities as it related to several features of metabolic syndrome. A similar biomarker profile is observed during preeclamptic pregnancies[14] and apparently persists into the fifth decade of life. Inadequate vascularization of several organs was recently substantiated in HFpEF by blunted vasodilator responses in myocardium,[43] skeletal muscle [44] and gluteal fat[45].

Comorbidities such as diabetes mellitus and chronic kidney disease were identified as drivers of myocardial remodelling in preclinical diastolic LV dysfunction and HFpEF [11, 13, 46] resulting in a specific cardiac phenotype[13]. Presence of diabetes raises LV mass and causes a larger reduction in LV diastolic distensibility[47, 48]. The present study observed similar features in patients with a history of early-onset preeclampsia, who also had larger LV mass and higher E/E’mean ratio at unchanged LV end diastolic volume index. Presence of chronic kidney disease also affects phenotypic appearance as it worsens cardiac mechanics evident from lower E’[49, 50]. Lower E’mean was also observed in the present study in women with a history of preeclampsia. In chronic kidney disease, effects were attributed to low myocardial microvascular density because of elevated sFlt-1, an antiangiogenic factor prominently involved in preeclampsia[51]. Future comparison of preclinical diastolic LV dysfunction in patients with and without history of preeclampsia will reveal if preeclampsia also leads to a distinct cardiac phenotype. Development of a specific preeclampsia-related phenotype could be the subject of future longitudinal follow-up of the current cohorts.

In conclusion, early-onset preeclampsia is associated with worse diastolic LV function and higher prevalence of preclinical diastolic LV dysfunction in the fifth decade of life. The association persists after accounting for age, smoking status, systolic blood pressure, BMI and educational level. Worse diastolic LV function is accompanied by higher plasma levels of IL-6 and sICAM-1, which are known to be elevated during preeclampsia. The shared biomarker profile may suggest insufficient vascularization because of systemic inflammation and endothelial dysfunction to be involved in the uterus at the time of pregnancy when developing preeclampsia and in the myocardium in the fifth decade of life when presenting with preclinical diastolic LV dysfunction.

## Abbreviations

A: late diastolic mitral flow velocity
BMI: Body mass index
E: Early diastolic mitral flow velocity
E’mean: Mean mitral annular lengthening velocity
HFpEF: Heart failure with preserved ejection fraction
hsCRP: high-sensitivity c-reactive protein
IL-6: Interleukin 6
LA volume index: Left atrial volume indexed by body surface area
LV: Left ventricular
LVEF: Left ventricular ejection fraction
MV dec time: deceleration time of the mitral valve
PIGF: Placental growth factor
sE-selectin: Soluble endothelial selectin
sFlt1: Soluble fms-like tyrosine kinase-1
sICAM-1: Soluble intercellular adhesion molecule-1
TNF-α: tumor necrosis factor alpha
